# Contextual interference effects on volleyball serve acquisition: a controlled trial with physical-education majors

**DOI:** 10.3389/fpsyg.2025.1635207

**Published:** 2025-08-04

**Authors:** Luping Qu, Wenxian Xiao, Shun Zhang, Ke Wang, Hui Li

**Affiliations:** ^1^School of Sports Science, Qufu Normal University, Qufu, Shandong, China; ^2^Nankai Primary School, Tianjin Central Ecological City, Tianjin, China

**Keywords:** contextual interference, volleyball serving technique, motor skill acquisition, teaching effectiveness, physical education students

## Abstract

To address limitations in previous domestic and international studies on “the effect of background interference on volleyball learning“, the present study selected 60 students majoring in physical education from the 2021 class at Qufu Normal University as experimental subjects to examine their skill acquisition. This study aimed to explore the differential effect of background interference on the learning of volleyball serving technique of students majoring in physical education. During an 8-week teaching experiment, the effects of three training modes, namely group block practice (low interference), mixed practice (medium interference), and random practice (high interference), on the learning effect of the serving technique of 60 physical education majors were compared. The results showed that (1) the block practice group performed optimally during the skill formation stage; (2) the random practice group had a significant advantage in the skill transfer test; and (3) the mixed practice group had the best effect in the long-term retention stage. The study provides empirical evidence for optimizing the teaching methods of volleyball techniques and has practical significance for the implementation of the policy of integration of sports and education.

## 1 Introduction

Physical and motor skills are defined as coordinated movement patterns governed by biomechanical principles and motor control theories, which are developed through systematic training protocols. These skills are typically acquired through structured physical education curricula, where they are refined and consolidated via deliberate practice regimens characteristic of athletic training. This developmental process holistically integrates students’ physiological capabilities, cognitive engagement, and psychomotor adaptation (He et al., 2022; Luo, 2022). Complete motor skill learning usually consists of three phases: motor skill mastery, skill retention and skill transfer, with mastery referring to the phase in which the individual repeatedly practices the task; retention referring to the phase in which the behavior of the previously learned information is maintained; and transfer referring to the phase in which the individual demonstrates an improvement in his ability to perform the different task variations (Prado et al., 2017; Emanuel et al., 2008).

With the development of international volleyball and the deepening of the reform of China’s volleyball program, volleyball has gained wide attention and has become one of the main teaching contents of physical education in colleges and universities. Through systematic learning of volleyball techniques, students can not only promote their physical growth and development, as well as enhance physiological functions and overall physical fitness, thereby effectively achieving the targeted learning objectives in the domain of physical health but also utilize this process as an essential method to maintain motivation and strengthen team cohesion. In the sport of volleyball, the serve is one of the most important techniques. It is also the beginning of the volleyball game, which is regarded as the “first tactical attack” of the whole team in the true sense of the word. The effect of the serve directly affects the effect of the opponent’s attack and the quality of tactical execution, which plays a decisive role in the game performance of the sports team (Drikos and Vagenas, 2011; Silva et al., 2014). Volleyball serve mainly has three ways: jump serve vigorously, jump serve floating ball, and stand serve floating ball, and the effect of serve technique has a significant correlation with several factors such as technical characteristics, ball speed and direction, team type and number of serving errors (Quiroga et al., 2012). Currently, studies on volleyball serving technique primarily focus on the serving type, serving line, and serving role (Pekyavaş et al., 2021), as well as their research involving the corresponding serve-receiving techniques, such as the type of serve-receiving technique used by the receiving player. The majority of studies are descriptive, analyzing the frequency of occurrence of different serve types and the number and frequency of various technical effects. However, there is a lack of research on introducing factors such as relevant contextual interference effects, which can promote the improvement of the volleyball skill level of practitioners.

The contextual interference effect, also known as the correlation intervention effect (Ren, 2019), refers to the introduction of interference factors by arranging the sequence of different skill practices when learning multiple motor skills, thus promoting the improvement of skill operation level of the practitioners (Wang and Dou, 2024). It was first proposed and defined by William Bartin as interference due to the context in which a skill is practiced, stating that different sequences of task performance or different practice conditions have different effects on skill learning, explaining the learning effects of practitioners under different practice conditions (Qu et al., 2020). Shea and Morgan first applied contextual interference to motor skill learning and arranged subjects to perform three different levels of difficulty in an arm-toss tennis practice experiment (Shea and Morgan, 1979). Low background interference, i.e., block training, is when skill practice is performed according to a designed training program that emphasizes focusing on the same skill or movement pattern over a short period of time. This type of training has significant advantages in the short-term skill acquisition phase, helping athletes establish stable movement patterns more quickly and reducing the error rate in the early stages of practice. Examples include AAA, BBB, CCC, and so on.

Medium background interference refers to the practice of multiple modalities in a fixed order, for example, ABC, ABC, ABC—and this mode of practice is usually referred to as mixed practice. High contextual interference refers to randomly arranged exercise procedures with high exercise variability, which may hinder the training effect and even exacerbate the trainer’s learning difficulty at the beginning of the training period but induces deeper information processing in the trainer, which leads to superior long-term retention effects and transfer performance. Examples include ABC, CBA, BAC, CAB, and so on (Qu et al., 2023; Aiken and Genter, 2018).

Relevant studies have shown that the low-interference group outperformed the medium- and high-interference groups in the skill acquisition phase, but the medium- and high-interference groups outperformed the low-interference group in the skill retention and transfer phases (Qu et al., 2020). However, Porter and Magill (2010), Dennis Landin, and other scholars in the study of background interference on the hand-held photoreceptor continuous tracking light experiments (Landin and Hebert, 1997), as well as 2 years of experience in college basketball shooting practice experiments, have confirmed that (progressive intensity) moderate background interference is more conducive to the learning and mastery of sports skills. In addition, other experiments have shown that whether or not a subject has a sports foundation affects the emergence of the background interference effect (Yu and Wen, 2018). Lin et al. (2008) used a joystick experiment in which subjects were trained to perform manual descriptions after viewing oscilloscope waveforms, and transcranial magnetic stimulation was performed to interfere with cortical motor areas between action tasks. The experiment demonstrated microscopically that high-intensity background interference strengthened the excitability of cortical motor areas and thus was more conducive to action learning. A neurophysiological basis was provided for the present experiment. The above studies have shown that the background interference effect is widely researched for different stages of technical learning in different sports. However, the existing studies have shown that although there is some involvement in the influence of background interference on volleyball serve learning of physical education students, they primarily focus on the short-term influence of single interference factors on serve technique and lack in-depth exploration of the mechanism. Based on this, this study will comprehensively consider a variety of background interference factors and include an in-depth discussion of how low, medium and high background interference affects the learning effect of volleyball serve technique, aiming to address the gap of previous studies in this field as well as to provide more comprehensive and targeted theoretical support for volleyball teaching and training.

Based on this, the present study assigned participants to block, mixed, and random practice groups, exposed each group to low-, medium-, and high-level background interference, and compared their learning effects during skill formation, skill retention, and skill transfer to examine the effects of different background interference on the learning effects of different phases of motor skills, as well as the effects of different background interference on the learning effects of different serving techniques in volleyball. In the study, two hypotheses were formulated. Hypothesis 1: Background interference (high interference, medium interference, low interference) has different effects on different stages of volleyball serve technique learning, in which the block group outperforms the mixed and random groups in the skill formation stage, while the mixed and random groups outperform the block group in the skill retention and transfer stage(). Hypothesis 2: The background interference (high interference, medium interference, and low interference) has different effects on specific techniques of volleyball serving, such as the frontal underhand serve, frontal overhand serve, and frontal overhand serve. Among them, the frontal underhand serve had the best effect, and the frontal overhand powerful serve and frontal overhand floating serve techniques had similar learning effects. The selection of the above three specific serving techniques was finalized by reviewing volleyball-related materials, according to the requirements of the general volleyball syllabus of Qufu Normal University, and combined with the actual situation of the students, as well as interviews with relevant experts. Among the three techniques, the underhand serve has a simple movement structure, the overhand powerful serve requires higher coordination, and the overhand floating serve is more difficult and involves finer hitting skills and touch point control. These three serve as forms of difference in movement complexity, neuromuscular coordination requirements, and environmental adaptations, providing an ideal skill vehicle for investigating contextual interference effects. Based on Gentile’s (Gentile, 1972) learning stage model and the hypotheses proposed by Guadagnoli and Lee (2004), as well as the results of the experiments conducted by Zhang (2017) and Zheng (2018) on the effects of background interference on the learning effects of basketball teaching experiments and tennis serving techniques, the anticipatory block practice group is more conducive to the learning and mastery of volleyball serving movement skills, the randomized practice group is more conducive to movement skill transfer, and the mixed practice group is more conducive to the long-term consolidation and improvement of the movement.

## 2 Experiment

For beginners, the volleyball program has more types of serving techniques that are more difficult for students to learn. The traditional teaching method, represented by repetition training, is a common method for learning movement skills, where practitioners repeat a movement to achieve power stereotypes. This method enables learners to focus on a specific movement without interruption, allowing them to develop good skill performance in the early practice stages. However, in terms of long-term mastery, this method is easy to lead to the athlete’s fatigue, which is not enough to meet the needs of the new era of student volleyball learning and cultivating quality talents. Background interference occurs when an individual learns multiple movement skills simultaneously, under various practice arrangements and organizational forms, leading to cross-task interference. It will be on the learner’s movement skills in the complex scenarios to produce a different degree of adaptability and operating level and will be more conducive to the learning of volleyball sports skills of students majoring in physical education (Jia, 2021). Therefore, the purpose of this experiment is to apply background interference to students’ volleyball serve movement technology learning, to investigate the influence of different background interference on the learning effect of the three volleyball serving techniques in the skill formation, retention and transfer stages, and to provide an actionable practical method for physical education teachers to teach volleyball skills and for students to learn volleyball skills. Based on a comprehensive analysis of the impact of background interference on the learning effect of volleyball serving techniques and the pre-experiment, combined with the actual situation of volleyball sports, the teaching process is comprehensively designed, and the above two hypotheses are put forward.

## 3 Materials and methods

### 3.1 Participants

Three general volleyball classes of physical education majors of Qufu Normal University in the class of 2021 (2022–2023 academic year) were selected as the subjects of the experiment. Each participant signed an experimental informed consent form. The participants indicated that they were beginners in volleyball. This means that they did not play volleyball in high school, did not participate in volleyball activity classes, did not participate regularly in recreational tournaments, and did not receive formal instruction on how to serve the ball. The students’ physical fitness, basic volleyball skills and other indicators were tested before the start of the experiment (the final number of students in each practice class was controlled at 20 students per class and an equal number of male and female students) to ensure that there were no significant differences between the experimental classes.

### 3.2 Pre-testing

Before the start of the formal experiment, the students’ height, weight, physical fitness and other indicators were prepared for testing. The homogeneity test, one-way ANOVA, and multiple comparisons of the height and weight of the subjects in each group yielded a *p* > 0.05, with no significant difference between the groups, satisfying the conditions for the conduct of the experiment (see Tables 1, 2). According to the characteristics of the volleyball program, access to relevant information (Jin, 2004; Huang, 2005), consulting and interviewing experts with rich experience in teaching volleyball, selecting three indexes of standing long jump, badminton throw and half-meter zigzag movement to test the physical fitness of the subjects, and carrying out the test of variance, there was no significant difference of *p* >0.05 (see Table 3).

**Table 1:** Basic information about the height and weight of the subjects.

Groups	Height (cm)	Weight (kg)	Quorum
Block exercise set	1.7833 ± 0.1367	66.4310 ± 19.7191	20
Mixed exercise set	1.7823 ± 0.1377	68.9809 ± 20.9810	20
Randomized exercise set	1.7762 ± 0.1562	65.9524 ± 22.0476	20

**Table 2:** Multiple comparisons of height and weight before formal experimentation.

	Classes	Groups	Significance
Height (m)	Block exercise set	Mixed exercise set	0.923
	Block exercise set	Randomized exercise set	0.727
	Mixed exercise set	Randomized exercise set	0.656
Weight (kg)	Block exercise set	Mixed exercise set	0.295
	Block exercise set	Randomized exercise set	0.966
	Mixed exercise set	Randomized exercise set	0.315

**Table 3:** Multiple comparisons of subjects’ basic athletic qualities before the formal test.

Projects	Classes	Groups	Significance
Badminton toss	Block exercise set	Mixed exercise set	0.322
	Block exercise set	Randomized exercise set	0.78
	Mixed exercise set	Randomized exercise set	0.476
Semicircular moving	Block exercise set	Mixed exercise set	0.809
	Block exercise set	Randomized exercise set	0.245
	Mixed exercise set	Randomized exercise set	0.162
Standing long jump	Block exercise set	Mixed exercise set	0.848
	Block exercise set	Randomized exercise set	0.569
	Randomized exercise set	Mixed exercise set	0.705

### 3.3 Apparatus and task

All participants were tested at the volleyball gym of the College of Sports Science, Qufu Normal University. The tasks practiced were three volleyball serving techniques: the frontal underhand serve, the frontal overhand serve, and the frontal overhand float serve technique.

### 3.4 Procedure

**Figure 1:**
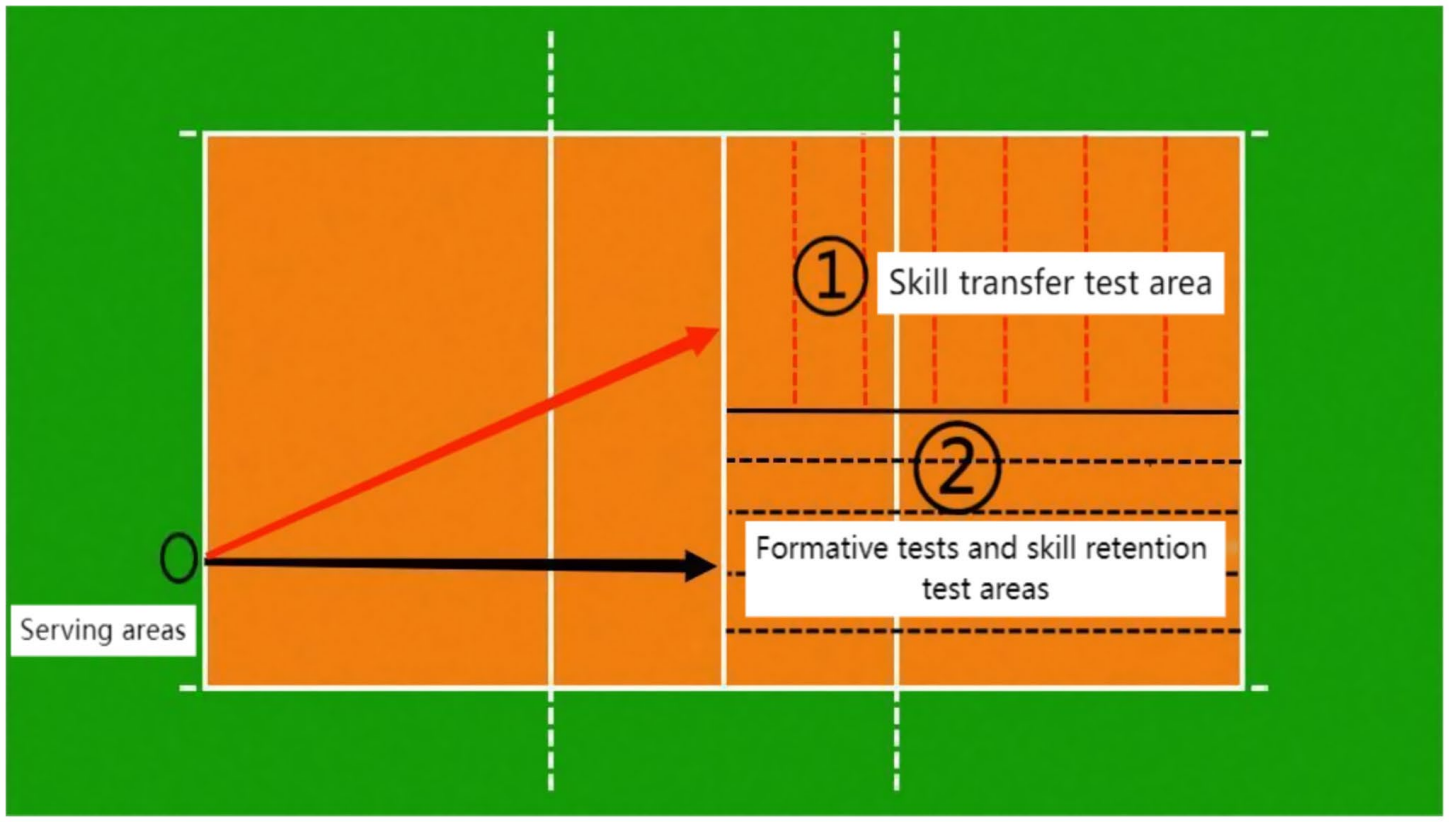
Volleyball serve test route.

To ensure the scientific validity and reliability of the experimental results, students in the three experimental classes were selected for this study according to the purpose of the experiment, which had certain similarities in age, gender, and exercise experience, which could reduce the potential influence of individual differences on the experimental results, and at the same time, a triple-blind experimental design was used to ensure the randomness of the groupings. First, all participants in the three experimental classes were numbered and equally assigned to the block exercise group (low interference), the mixed exercise group (medium interference), and the random exercise group (high interference) by a computer-generated random number sequence. The number of people in each practice group and the distribution of male and female students should be approximately equal to ensure that there is no significant difference in the number of students of different genders in each group. Throughout the randomization process, an independent research assistant will be responsible for operating the random number generator and recording all information during the randomization process, including participant numbers, random numbers, and group results. These records will be kept properly for verification and validation in subsequent studies. All participants read the same step-by-step instructions that explained the steps of the exercise and how to properly complete the three tasks. The researcher gave a short demonstration of each session. Participants were told to stand behind the serving line and perform the three serving techniques (see Figure 1). They were also told to move in a standardized manner, hit the ball at the exact spot, coordinate their body power, and land serves accurately. Each type of serve was given five chances, with four points awarded for a serve into the designated area, two points for a serve into the court but outside the designated area, and no points for a missed serve. The full score for each type of serve was 20 points. In order to further verify the validity and mutual trust of the assessment criteria, this study trained and assessed the assessment team before the experiment. The training included a detailed explanation of the assessment criteria, a description of the scoring rules, and a demonstration of the actual scoring operation. During the appraisal process, the members of the assessment team scored a set of pre-experimental data independently, and then the scoring results were tested for consistency. The results showed a high degree of consistency in the scores of the assessment team members, indicating that the assessment criteria have good validity and mutual trust.

The test at the end of each experiment is in the form of teaching and testing separation, and the assessment criteria for the student skill test are a combination of quantitative and qualitative evaluation. Quantitative evaluation: 4 points were awarded for serving within the designated area; otherwise, no points were awarded, and five balls for each type of serve were scored out of 20 points. Qualitative evaluation: The study invited two experts with rich experience in volleyball teaching and training to form an evaluation team. The expert panel held discussions and consultations before the experiment and formulated detailed assessment criteria and scoring rules (see Table 4). The assessment criteria covered all aspects of serving techniques, including movement normality, power control, accuracy, and so on. Specifically, according to the scoring rules, the experts scored the students on the technical completion of the movement and took the average score out of 5 points. To ensure the fairness of the scoring, the scoring results of all evaluators were calibrated and adjusted at the end of the experiment.

**Table 4:** Serve movement skill scoring criteria.

Movement evaluation criteria	Score
Standardized movement, accurate hitting area, coordinated body force, and accurate landing point	5
More standardized movement, more accurate hitting area, coordinated body force, and accurate landing point	4
Average movement specification, more average hitting area, more coordinated body force, and more accurate landing point	3
Fairly standardized movements, inaccurate hitting area, general coordination of body force generation, and inaccurate landing point	2
Unreasonable movement specification, inaccurate hitting part, uncoordinated body force, inaccurate landing point	1

After the collection of the test data, a rigorous and transparent process was adopted for statistical analysis in this study section. First, data cleaning and preprocessing were performed on the collected raw data, including the elimination of outliers and the handling of missing data. Second, before ANOVA was performed, the data were tested for normality and variance chi-square to ensure that the data met the basic assumptions of ANOVA. Data that did not satisfy the assumptions were analyzed using the appropriate data transformation methods or non-parametric statistical methods.

### 3.5 Practice

Practice schedule and practice volume (Table 5). Briefly, each participant completed 540 practice trials (6 weeks total, two sessions per week, 12 sessions total, 45 serves per session). The block practice group performed 180 serves of Exercise A in the first and second weeks, 180 serves of Exercise B in the third and fourth weeks, and finally 180 serves of Exercise C in the fifth and sixth weeks; the mixed practice group performed 135 serves of Exercise A in the first session of the first and second weeks, 135 serves of Exercise B in the second session of the second week and the third week, 135 serves of Exercise C in the first session of the fourth and fifth weeks, and 135 serves of Exercise C in the fifth The second session and the sixth week performed all three ABC serves (15 serves of each) 135 times per session; the randomized practice group practiced all serves randomly (guaranteeing 15 serves of each type per session, 45 serves per session, and equal totals).

**Table 5:** Organization of course exercises.

T G	Week 1		Week 2		Week 3		Week 4		Week 5		Week 6	
	L1	L2	L1	L2	L1	L2	L1	L2	L1	L2	L1	L2
Block Exercise Set	A	A	A	A	B	B	B	B	C	C	C	C
Mixed Exercise Set	A	A	A	B	B	B	C	C	C	ABC	CBA	BCA
Randomized Exercise Set	ABC	CBA	CAB	BAC	ACB	BCA	BAC	ABC	CAB	CBA	CBA	BCA

① Serve from the front underhand, ② forehand overhand, ③ and forehand drifting ball.

### 3.6 Formation, retention, and transfer tests

In the formation and retention tests, the participants stood at the serving position and performed the test serve in a straight line, as shown in Figure 1, following a qualitative and quantitative approach, with five serves for each type of serve, four points for serves into the designated area, two points for serves into the court but outside the designated area, and no points for missed serves out of a total of 20 points. Following the retention test, participants stood behind the serving line near the side position and were tested on the serve using the diagonal line approach in Figure 1. Scoring was again conducted using a combination of qualitative and quantitative methods, with each subject being tested on the relocation of three types of serves, with five chances for each type of serve, four points for serves to the designated area, two points for serves to the court but outside the designated area, and no points for misses of serves, with each type of serve being 20 points.

## 4 Results

### 4.1 General characteristics of contextual interference on motor skill learning

Total performance of different subject groups at different stages after the experiment (see Table 6).

**Table 6:** Overall performance of the experimental class at each stage after the experiment.

GroupsTesting phase	Formative phase	Retention phase	Transfer phase
Block exercise set	1,307	1047.7	951.2
Mixed exercise set	1028.6	1246.1	1095.7
Randomized exercise set	1144.2	1124.2	1173.7

It can be seen that, in terms of the formation test stage, the best performance is that of the block practice group, followed by the random practice group; the worst performance is that of the mixed practice group. In terms of the skill retention test stage, the mixed practice group has the best performance, followed by the random practice group, and the relatively worst performance is that of the block practice group. In terms of the skill transfer stage, the best performance is that of the random practice group, followed by the mixed practice group, and the relatively worst performance is that of the block practice group. The group’s performance was the best, the mixed practice group’s performance was the second best, and the block practice group’s performance was the worst in relative terms.

### 4.2 Experimental results on the influence of different background interferences on the effects of different stages of motor skills learning

The stage test data obtained during the experiment were entered into an Excel 2019 worksheet, and according to the purpose of the study, the Excel 2019 sheet was used in the preliminary stage to carry out the corresponding statistics. The statistical data were rearranged and analyzed using SPSS Statistics 25.0 to carry out descriptive statistics, the variance chi-square test, one-way ANOVA, and multiple comparisons analysis.

#### 4.2.1 Experimental effects of different background interference groups on the formative stages of motor skill learning

(1) Serve underhand

A comparison of frontal underhand serves under low, medium, and high background interference reveals that the block group (22.95 ± 2.68) outperformed the random group (20.28 ± 3.58) and the mixed group (18.91 ± 3.14). The data were tested to show a normal distribution pattern with a variance of 8.488 between the groups, which has a significance value of less than 0.001 (i.e., *p* < 0.01), showing a significant difference (see Table 7). Multiple comparisons between all groups revealed a significant difference (*p* < 0.01) between the block and randomized groups. Additionally, the mixed and randomized groups showed no difference (*p*-value = 0.176, *p* > 0.05). It can be concluded that the volleyball frontal underhand serve technique block group has the best performance, followed by the mixed group, and the worst is the randomized group, but the randomized group and the mixed group have similar performance.

(2) Forehand overhand

**Table 7:** Analysis of the technical test of the front-hand underhand serve in the skill formation phase.

Groups	*N*	X ± S	Variance chi-square test	one-way ANOVA	
			*P*	*F*	Sig
Block exercise set	20	22.95 ± 2.68	0.507	8.488	0.001
Mixed exercise set	20	18.91 ± 3.14			
Randomized exercise set	20	20.28 ± 3.58			

The block group (21.29 ± 3.50) performed better than the mixed group (16.55 ± 3.87) & random group (18.55 ± 4.04) in volleyball frontal overhand power shots under low, medium & high background interference. The data were tested to show a normal distribution pattern with a significant difference in *p*-value (i.e., *p* < 0.01) (see Table 8). Multiple comparisons were made between all the groups. The *p*-value of *p* < 0.01 indicated a significant difference between the block and mixed groups. In contrast, the *p*-value of 0.101 (*p* > 0.05) did not exist for the randomized and mixed groups when compared to each other. It can be concluded that the best performance was in the block group, followed by the mixed group, and the worst was in the randomized group, which was closer to the performance of the mixed group.

(3) Forehand drifting ball

**Table 8:** Analysis of the frontal overhand power shot test in the skill formation stage.

Groups	*N*	X ± S	Variance chi-square test	one-way ANOVA	
			*P*	*F*	Sig
Block exercise set	20	21.29 ± 3.50	0.741	7.832	0.001
Mixed exercise set	20	16.55 ± 3.87			
Randomized exercise set	20	18.55 ± 4.04			

Comparing the volleyball frontal overhand serve under low, medium, and high background interference, it can be concluded that the performance of the block group (21.115 ± 3.4826) was better than the mixed group (15.975 ± 3.9939) and better than the random group (18.385 ± 3.9625). The data were tested to conform to the law of normal distribution, with a highly significant difference at *p* < 0.01 (see Table 9). Multiple comparisons were made between all the groups, and the *p*-value of the performance between the block group and the mixed group was *p* < 0.01, showing a significant difference, while the *p*-value of the randomized group and the mixed group was 0.051 (*p* > 0.05), not showing a significant difference.

**Table 9:** Skill formation stage frontal overhand drift test analysis.

Groups	*N*	X ± S	Variance chi-square test	One-way ANOVA	
			*P*	*F*	Sig
Block exercise set	20	21.12 ± 3.48	0.664	9.063	0.000
Mixed exercise set	20	15.98 ± 3.99			
Randomized exercise set	20	18.39 ± 3.96			

The above study showed that in the skill formation stage, the best test scores for the three serving styles of volleyball were in the block group, followed by the mixed group, and the worst was the random group.

#### 4.2.2 Experimental effects of different background interference groups on the retention phase of motor skill learning

Comparing the frontal underhand serves under low, medium, and high background interference, it can be concluded that the mixed group (21.37 ± 3.67) outperformed the random group (20.07 ± 3.62) and the block group (17.96 ± 3.07). After that, multiple comparisons between all the groups yielded a *p*-value of 0.003 (*p* < 0.01) for a highly significant difference between the block group compared to the mixed group and a p-value of 0.242 (*p* > 0.05) for the mixed group compared to the randomized group, which was not significant. This leads to the conclusion that the mixed group had the best performance, followed by the randomized group, and the worst was the block group (see Table 10).

**Table 10:** Skill retention phase frontal underhand serve test analysis.

Groups	*N*	X ± S	Variance chi-square test	one-way ANOVA	
			*P*	*F*	Sig
Block exercise set	20	17.96 ± 3.07	0.497	4.917	0.011
Mixed exercise set	20	21.37 ± 3.67			
Randomized exercise set	20	20.07 ± 3.62			

Comparison of frontal overhand power serve under low, medium, and high background interference shows that the mixed group (20.21 ± 3.4537) outperformed the random group (18.36 ± 4.0419) and the block group (17.32 ± 2.6253). Afterward, multiple comparisons between all the groups yielded a *p*-value of 0.01 as compared to the block group and the mixed group with a highly significant difference, a *p*-value of 0.341 as compared to the randomized group with no significant difference, and a *p*-value of 0.093 as compared to the mixed group and the randomized group with no significant difference (see Table 11).

**Table 11:** Skill maintenance phase frontal overhand power ball technical analysis.

Groups	*N*	X ± S	Variance chi-square test	One-way ANOVA	
			*P*	*F*	Sig
Block exercise set	20	17.32 ± 2.63	0.163	3.657	0.032
Mixed exercise set	20	20.21 ± 3.45			
Randomized exercise set	20	18.36 ± 4.04			

Comparing the frontal overhand serve under low, medium, and high background interference, it can be concluded that the mixed group (20.73 ± 3.05) outperformed the random group (17.18 ± 3.52) and the block group (17.11 ± 3.18), and the data were tested to show a normal distribution (see Table 12). Subsequent multiple comparisons among all the groups revealed that the mixed group performed the best, followed by the randomized group, and the block group performed the worst.

**Table 12:** Skill retention stage overhand drift technical analysis.

Groups	*N*	X ± S	Variance chi-square test	One-way ANOVA	
			*P*	*F*	Sig
Block exercise set	20	17.11 ± 3.18	0.469	7.011	0.002
Mixed exercise set	20	20.73 ± 3.05			
Randomized exercise set	20	17.78 ± 3.52			

The above study showed that in the skill retention phase, the mixed group was the best in the three serving styles test performance in volleyball, followed by the random group, and blocked practice ranked last.

#### 4.2.3 Experimental effects of different background interference groups on the stage of motor skill learning transfer

The analysis of the migration phase of the test showed that the frontal underhand serve under low, medium, and high background interference, the random group (20.57 ± 3.16) outperformed the mixed group (18.71 ± 3.29) and the block group (16.77 ± 3.10) in the descriptive statistics results, with the data being normally distributed. Subsequent multiple comparisons between groups yielded the best performance for the random group, followed by the mixed group, and the worst for the block group (see Table 13).

**Table 13:** Skill transfer stage frontal underhand serve technique test analysis.

Groups	*N*	X ± S	Variance chi-square test	One-way ANOVA	
			*P*	*F*	Sig
Block exercise set	20	16.77 ± 3.10	0.811	7.12	0.002
Mixed exercise set	20	18.71 ± 3.29			
Randomized exercise set	20	20.57 ± 3.16			

Analysis of the migration phase of the test showed that the random group (18.285 ± 3.84) performed better than the mixed group (18.085 ± 3.4095) and the block group (15.29 ± 2.9374) in the frontal overhand serve of the power ball under low, medium and high background interference, and the data were normally distributed. Subsequent multiple comparisons among the three groups showed that there was a significant difference between the block group and the mixed group with a *p*-value of 0.012 (*p* < 0.05), and there was no difference between the mixed group and the random group with a *p*-value of 0.854 (*p* > 0.05); thus, it can be seen that the random group had the best performance, followed by the mixed group, and the worst was the block group (see Table 14).

**Table 14:** Analysis of overhand power ball technical tests in the skill transfer phase.

Groups	*N*	X ± S	Variance chi-square test	One-way ANOVA	
			*P*	*F*	Sig
Block exercise set	20	15.29 ± 2.94	0.107	4.807	0.012
Mixed exercise set	20	18.09 ± 3.41			
Randomized exercise set	20	18.29 ± 3.84			

The results of descriptive statistics showed that the random group (19.83 ± 3.77) outperformed the mixed group (18.00 ± 3.46) and the block group (15.50 ± 3.00) under the low, medium, and high background interference with the frontside overhand drifting. The data were normally distributed with a *p-*value of *p* < 0.01, showing a highly significant difference. Afterwards, multiple comparisons between groups were performed, with a *p*-value of 0.025 (*p* < 0.05) showing a significant difference between the block and mixed groups, and a *p* value of *p* < 0.01, showing a highly significant difference between the randomized group and the block group. This shows that the randomized group performed the best, followed by the mixed group, and the worst was the block group (see Table 15).

**Table 15:** Skill transfer stage overhand drift technical test analysis.

Groups	*N*	X ± S	Variance chi-square test	One-way ANOVA	
			*P*	*F*	Sig
Block exercise set	20	15.50 ± 3.00	0.332	8.051	0.011
Mixed exercise set	20	18.00 ± 3.46			
Randomized exercise set	20	19.83 ± 3.77			

The results of the above data supported the first hypothesis, proving that different background interference had different effects on the learning effects at different stages of college students’ volleyball serve technique learning. The block group performed the best in the skill formation stage (95% CI [79.1,85.5]), which was 18.7% higher than that of the randomized practice group (Cohen’s *d* = 1.32, *p* < 0.001). The reason for this can be analyzed because, in the early stage of motor skill acquisition, the low background interference practice method enables students to perform a large number of single-movement exercises in a short period, allowing them to build a representation of the movement quickly. While in the stage of skill retention and transfer, the mixed and randomized groups outperformed the block groups.

### 4.3 The effect of different background distractions on the learning effect of different serving techniques

In order to understand the effect of different background interference situations on different serving techniques, the data were rearranged and analyzed using SPSS Statistics 25.0 for descriptive statistics, chi-square test, one-way ANOVA, and multiple comparisons.

#### 4.3.1 The effect of low background interference on the learning outcomes of three serving techniques

Low background interference of the three serving techniques in the skill formation stage, frontal underhand serve performance (22.95 ± 2.68) was better than frontal overhand serve with power (21.29 ± 3.498) and frontal overhand serve with drift (21.12 ± 3.48). The data were normally distributed. Analysis of the retention-phase test showed that the frontal underhand serve (17.96 ± 3.07) outperformed both the frontal overhand powerful serve (17.32 ± 2.63) and the frontal overhand floating serve (17.11 ± 3.18), and the data conformed to the law of normal distribution. Analysis of the test in the migration phase showed that the frontal underhand serve (16.77 ± 3.10) was better than the frontal overhand serve (15.50 ± 3.00) and the frontal overhand serve (15.29 ± 2.94), and the data were normally distributed. It can be seen that low background interference had no significant effect on the learning effect of the different serving techniques across stages.

#### 4.3.2 The effect of mid-context interference on the learning of three serving techniques

The results of the middle background interference on the three serving techniques tested showed that the frontal underhand serve performance (18.91 ± 3.14) was better than the frontal overhand powerful serve (16.55 ± 3.87), which was better than the frontal overhand floating serve (15.98 ± 3.99). The data were tested to be normally distributed with a *p*-value of 0.035 (*p* < 0.05) for a significant difference. Afterwards, multiple comparisons were performed and found that the P of 0.047 (*p* < 0.05) for the underhand serve and the overhand power serve showed significant differences; the *p* value of 0.627 (*p* > 0.05) for the overhand power serve compared to the overhand floater did not show significant differences. At this stage, the medium background interference had the best effect on the learning effect of the volleyball front-hand underhand serve technique.

#### 4.3.3 The effect of high background interference on the learning effect of three serving techniques

The results of high background interference on the test of the three serving techniques showed that the frontal underhand serve (18.71 ± 3.29) outperformed the frontal overhand powerful serve (18.09 ± 3.41), which in turn outperformed the frontal overhand floating serve (17.96 ± 3.46). The data were tested to be normally distributed with a *p*-value of 0.133 (*p* > 0.05). There was no significant difference and no statistical significance. However, multiple comparisons were conducted and found that there was a significant difference between the underhand serve and the overhand power serve; the *p*-value of the underhand serve and the overhand floating ball was 0.188 (*p* > 0.05), and the *p*-value of the overhand power serve and the overhand floating ball was 0.826 (*p* > 0.05). There was no significant difference. The above analysis shows that the skill transfer stage is favorable for the learning of the volleyball underhand serve technique under high background interference.

The above analysis shows that the skill transfer stage favors the learning of the volleyball underhand serve technique under high background interference. Therefore, these data results validate Hypothesis 2: varying background interference affects students’ learning of volleyball serving techniques at different stages. The frontal underhand serve has the best effect, while the learning effects of the frontal overhand powerful serve and the frontal overhand floating serve are similar.

## 5 Discussion

In this study, college students in the College of Physical Education were taken as the experimental subjects to learn the three motor skills of volleyball, namely, underhand serve, overhand powerful serve and overhand floating serve, and set up three practice groups with different background interference (low background, medium background and high background interference patterns), corresponding to which the practice groups were the block practice group, the mixed practice group and the randomized practice group, respectively. From the above analysis, it can be seen that, firstly, in the formation stage of motor skills, the comparative analysis of the subjects’ test scores of the three serving skills of volleyball shows that the different background interference methods of high, medium and low appeared to have different impact effects, and there is a significant difference. The students in the block group (low background interference) practiced better relative to the mixed group (medium background interference) and random group (high background interference), and there was a significant difference. This is consistent with the findings of Vera and Montilla’s (Vera et al., 2008) study on soccer skill acquisition, where block practice was used. That is, during the skill formation stage, the practice effect of the block group was superior to the learning effect of the randomized and mixed groups. It is worth noting that group block training also has irreplaceable advantages for beginner motor skill learners. By reducing the cognitive demands of task-switching, block training helps beginners focus their attention on the modification of a single movement parameter, such as the height of a volleyball serve, thus accelerating novice learners’ initial motor proficiency (Bonney et al., 2017).

Secondly, in the stage of the skill retention test, it was learned through the processing and analysis of the experimental data that the mixed group performed the best, the random group was the second best, and the block group performed poorly in that stage. This is in line with the results of Bai (2016), Wang (2013), Feghhi et al. (2011), and Song (2018), who conducted research on college students’ tennis, motor skills learning, basketball free throws, and soccer skills. Whereas, the best performance of the random group (high background interference), the second-best performance of the mixed group (medium background interference), and the worst performance of the block group (low background interference) were generally observed during the skill transfer phase. This is consistent with Kimiya Sadri, Hassan, and Mohammadzaden’s study (Sadri et al., 2013) on badminton serves, which concluded that the performance of the random group was better than the performance of the mixed and random groups during the skill transfer phase. This result is in line with the experimental expectations.

Finally, through the analysis of the experimental results, it was learned that different background interference has different interference effects. Specifically, under the low background interference, no significant difference was found in the learning of the three serving techniques; under the medium background interference, a significant difference was found in the formation stage, and the frontal underhand serve was the best; under the high background interference, a significant difference was found in the learning of the underhand serve technique, which was the best performance. Looking at the different phases, test scores showed that the frontal underhand serve technique was the best learned, both in the formation, retention, and transfer phase tests, a result that can be explained from the perspective of relevant movement theories. The movement complexity theory points out that the underhand serve, as a basic technique, relies mainly on the upper limb swing, has a relatively simple movement structure, and requires less working memory and movement adjustment in a background interference environment. In contrast, the frontal overhand serve and frontal overhand floater serve, two techniques that require precise coordination of multiple spatial and temporal parameters such as throwing, running, and hitting, are more prone to movement control breakdowns in a high interference condition (Druckman and Bjork, 1992).

The results of this study have important practical significance for volleyball teaching and training. In volleyball teaching, coaches can rationally arrange the background interference conditions according to the skill level and technical characteristics of the trainees to improve the teaching effect. For beginners, it is recommended to use low-interference group block practice in the skill formation stage to help them quickly establish a stable movement pattern. With the improvement of trainees’ skill level, the difficulty of background interference is gradually increased by adopting the mixed practice with medium interference and the random practice with high interference to enhance the long-term retention and transferability of skills. For example, in the general volleyball course, teachers can first arrange students to practice the repetition of a single serving technique and then introduce background interference conditions of different difficulty after the trainees have mastered the basic movements, such as playing background noises of different intensities and adjusting the position of the target area of the serve during the serve. Through this diversified practice, students can better master the serving technique in the complex and changing environment and improve the success rate and threat of the serve in the game. However, the results need to be used in the context of the individual when generalizing to experienced athletes and non-student groups. According to relevant studies, experienced athletes have established stable movement standards, and their resistance to interference may be significantly better than that of student subjects. In addition, professional athletes’ training situations are more idiosyncratic, and their serving performance is influenced by complex factors such as tactical awareness and match experience, which are somewhat different from laboratory-controlled training conditions. Therefore, it is recommended that subsequent studies examine the applicability of contextual interference to professional athletes in real training environments and consider combining technical and tactical training to better meet the practical needs of high-level athletes.

In addition to that, this study has certain novelties and contributions in the field of research on the effect of background interference on volleyball serve technique learning. First, it systematically explored the effects of different background disturbances (low, medium, and high disturbances) on the different stages (skill formation, skill retention, and skill transfer) of volleyball serve technique learning, which provided comprehensive empirical support for the application of background disturbance effects in volleyball. While the majority of the previous studies focused on descriptive analysis, this study delved into the specific mechanisms and influencing factors of the background interference effect through experimental design and statistical analysis. Second, the study selected three serving techniques with different technical complexity and neuromuscular coordination needs, namely, underhand serve, overhand powerful serve and overhand floating serve, as the research objects to provide a comparative analysis of the application of the background interference effect in the different types of techniques, which provides a theoretical basis for the design of the personalized training program.

## 6 Conclusion

In conclusion, the experiment verified the presence of background interference and proved that a background interference effect exists in real volleyball teaching, affecting beginners in the sport. Different practice methods lead to different effects. At the motor-skill formation stage, the block practice group (low background interference) is more suitable for learning motor skills; at the motor-skill retention stage, the mixed-practice group (medium background interference) is more conducive to motor-skill learning, and at the motor-skill transfer stage, random practice (high background interference) is more conducive to motor-skill learning. From the perspective of motor skill difficulty, the lower the difficulty level of the motor skill, the greater the degree of background interference. This experiment investigated the training of three serving styles in beginner volleyball players using changeover training, which induces a background interference effect and improves learning. These findings hold practical value for volleyball skill learning, and future research can build on this by applying background interference to other volleyball techniques or other sports. It can be verified whether background interference affects students within a given sport specialization and allows for comparisons across different specializations.

## 7 Research limitations

We must recognize that there are some limitations to this study. First, the experimental subjects were all students with no volleyball experience, which, although it helps to control for differences in basic skill levels, may limit the generalizability of the findings. The volleyball serve, as a technically demanding motor skill, may have differences in its learning curve and interference effects among athletes of different levels. Such differences may lead to different performance of the background interference effect. Second, the relatively short experimental intervention period failed to fully observe the long-term training effects, especially the higher-order abilities of skill retention and transfer. Future studies should include subjects with different skill levels, such as amateur volleyball players versus professional athletes, to stratify and compare the variability in the applicability of background interference. In addition, the duration of the intervention should be extended, and, at the same time, the background interference should be expanded to other volleyball skills, such as dunking and passing, to better serve the needs of real-world play.

## Data Availability

The original contributions presented in the study are included in the article/supplementary material. Further inquiries can be directed to the corresponding author/s.
